# Piezo1 promotes intervertebral disc degeneration through the Ca^2+^/F-actin/Yap signaling axis

**DOI:** 10.1186/s10020-025-01147-z

**Published:** 2025-03-08

**Authors:** Fushuai Peng, Mingtong Sun, Xingzhi Jing, Fei Chen, Tong Cao, Zhenzhen Li, Tao Li

**Affiliations:** 1 Department of Spine Surgery, Shandong Provincial Hospital Affiliated to Shandong First Medical University, No. 324, Jingwu Weiqi Road, Huaiyin District, Jinan, Shandong 250021 China; 2 Department of Emergency Surgery, Yidu Central Hospital of Weifang City, Weifang, Shandong 262500 China; 3 Department of Spinal Surgery, The Affiliated Hospital of Qingdao University, No. 59, Haier Road, Qingdao, Shandong 266075 China; 4 Department of Ultrasound, Shandong Provincial Hospital Affiliated to Shandong First Medical University, No. 324, Jingwu Weiqi Road, Huaiyin District, Jinan, Shandong 250021 China

**Keywords:** Intervertebral disc degeneration, Chondrocyte, Piezo1, Yes-associated protein, F-actin, Oxidative stress

## Abstract

**Background:**

Piezo1 is a mechanically sensitive cation channel expressed in various tissues of the human body and has multiple roles in both physiological and pathological processes. However, its role in the occurrence and development of intervertebral disc degeneration (IVDD) is not fully understood.

**Methods:**

In the present study, an IVDD mouse model and Piezo1 small interfering (si)RNA was used to investigate the role of Piezo1 in IVDD progression. Furthermore, the Ca^2+^ inhibitor, BAPTA-AM, and the F-actin cytoskeleton polymerization inhibitor, Latrunculin A, were employed to examine the roles of Ca^2+^ influx and cytoskeleton dynamics in Piezo1-mediated IVDD progression. Additionally, Yes-associated protein (Yap) small interfering (si)RNA was used to investigate the involvement of Yap in Piezo1-induced IVDD progression.

**Results:**

The findings of the present study indicated that Piezo1 was positively associated with IVDD and that Piezo1 upregulation promoted IVDD via facilitating cartilage endplate (CEP) degeneration and calcification. The Ca^2+^ inhibitor, BAPTA-AM, and the F-actin cytoskeleton polymerization inhibitor, Latrunculin A, inhibited Piezo1-mediated extracellular matrix degradation and CEP chondrocyte degeneration. Moreover, it was found that Piezo1 activated Yap through an F-actin-mediated non-canonical pathway and that Yap siRNA inhibited Piezo1 upregulation-induced IVDD progression.

**Conclusion:**

Overall, the results of the present study indicate that increased expression of Piezo1 is closely related to the occurrence and development of IVDD and that the Piezo1-mediated Ca^2+^/F-actin/Yap axis contributes to this process. Thus, targeting Piezo1 may provide a new strategy for the treatment of IVDD.

## Introduction

Intervertebral disc degeneration (IVDD) is the main cause of lower back pain and other intervertebral disc diseases (Khan et al. 2017). As age increases, the process of IVDD accelerates, exacerbating symptoms and significantly impacting the quality of life, while also imposing a substantial economic burden (Vergroesen et al. 2015). At present, there are four methods to treat IVDD: Non-invasive treatments such as drug therapy, multi-physical and multidisciplinary bio-psychosocial rehabilitation, interventional therapy and surgical treatment (Zhao et al. 2019). Compared with surgical treatment, drug treatment is milder, less harmful to patients and has a lower economic burden. However, the evidence supporting drug treatment is limited, highlighting the need to identify suitable drugs to slow IVDD progression by studying its causation.

The intervertebral disc consists of three parts: (i) The outer fibrous annulus, which is composed of thick fibrocartilage; (ii) the central nucleus pulposus (NP), a gel-like mass; and (iii) the cartilage endplate (CEP), a layer of hyaline cartilage located on the upper and lower surfaces of the vertebral body. This structure connects two adjacent vertebrae and primarily functions to maintain spinal stability (Roberts et al. 2006). As the largest non-vascular tissue in the human body, the intervertebral disc receives most of its nutrition from the CEP (Raj 2008). The integrity of the intervertebral disc is influenced by a number of factors, such as mechanical stress, trauma, heredity and nutrition. The upright posture of humans subjects the intervertebral disc to prolonged compressive stress, accelerating its degeneration (Kos et al. 2019). Both aging and mechanical stress induce oxidative stress and low-grade chronic inflammation in chondrocytes, increasing the production of reactive oxygen species (ROS) and pro-inflammatory cytokines such as IL-1β and TNF-α (Wang et al. 2016; Wang et al. 2021; Chen et al. 2024). These factors promote extracellular matrix (ECM) degradation and CEP calcification, leading to apoptosis, structural change, and further degeneration of the intervertebral disc, creating a vicious cycle (Zhang et al. 2020). Oxidative stress also has an important role in the pathogenesis of osteoarthritis (Loeser et al. 2016; Minguzzi et al. 2018). Notably, high levels of oxidative stress responses are observed in degenerated CEP (Han et al. 2019). Hydrogen peroxide, a common ROS, exacerbates oxidative stress in cells, primarily causing ECM degradation and CEP calcification (Yuan et al. 2019; Wang et al. 2018). Tert-butyl hydroperoxide (TBHP), as a derivative of hydrogen peroxide, also exhibits strong oxidizing properties similar to hydrogen peroxide. Consequently, the present study utilized TBHP to interfere with chondrocytes, simulating the process of IVDD to explore the potential mechanisms promoting IVDD.

The Piezo family includes two protein types, Piezo1 (Fam38A) and Piezo2 (Fam38B) (Coste et al. 2010). Piezo1 protein is found in numerous cell types and belongs to the mechanically sensitive ion channel family. These channels can detect various mechanical stimuli, including hydrostatic pressure, shear stress and membrane tensile stress, which can be directly or indirectly applied to the cell membrane through its surrounding components (Poole et al. 2014; Li et al. 2014). As an ion channel, Piezo1 allows the infiltration of various cations into the cell, particularly Ca^2+^ (Kim et al. 2023; Wang et al. 2023; Sugimoto et al. 2017). Upon mechanical stimulation, a significant influx of Ca^2+^ occurs, which acts as a signal to mediate multiple pathways in osteoblasts, osteocytes, and chondrocytes. This process triggers a series of mechanical reactions and regulates various cellular processes, such as proliferation, differentiation and apoptosis (Borbiro et al. 2017). Research has shown that Piezo1 serves an important role in Ca^2+^-dependent cell death, and its activation is associated with changes in cytoskeletal support and membrane structure (Kim et al. 2023; Koike et al. 2015; Griffin et al. 2005; Wang et al. 2022a, b). Piezo1 protein is highly expressed in mouse articular chondrocytes (Lee et al. 2014). Mechanical loading enhances the ability of chondrocytes to uptake Ca^2+^ and induces chondrocyte apoptosis; however, the addition of specific small interfering (si)RNA to reduce the expression of Piezo1 protein can reduce the mechanical stress-induced apoptosis of chondrocytes. In addition, GsMTx4, a pharmacological inhibitor of Piezo1, can also reduce the mechanical stress-induced apoptosis of chondrocytes (Lawrence et al. 2017). In recent years, research on Piezo1 protein has increased, highlighting its important role in promoting the apoptosis of articular chondrocytes and aggravating joint degeneration. Nevertheless, there are few studies on the specific role and mechanism of Piezo1 protein in the process of IVDD.

Yes-associated protein (Yap), an important mediator of the Hippo pathway, is a transcription factor that shuttles between the nucleus and cytoplasm. Yap affects the expression of multiple genes and regulates cell proliferation and apoptosis (Xiang et al. 2018). Related studies have confirmed that Yap also plays an important role in osteocytes, and the upregulation of Yap enhances the expansion and osteogenic differentiation of osteoblast precursors (Kegelman et al. 2021). Yap can be activated not only by oxidative stress, but also by mechanical stress stimulation (Wang et al. 2019). Human growth plate chondrocytes exist in a dynamic stress environment, where external mechanical signals are transmitted to the cells in different ways, such as affecting the expression of Piezo1 or the growth and depolymerization of cytoskeleton fibers (Lauer et al. 2021; Brylka et al. 2024). RhoGTPases are key regulators of cytoskeleton F-actin, and include RhoA, Rac1 and cell division cycle 42 (Kloc et al. 2019). Focal adhesions (FAs) are multi-molecular complexes located between integrin and cytoskeleton F-actin that mediate the ECM and cellular bone transduction process between filaments of F-actin (Dasgupta et al. 2019). Intervention with botulinum toxin C3, an inhibitor of RhoA, or Latrunculin B, an inhibitor of the cytoskeleton, can inhibit the dephosphorylation of Yap protein (Xu et al. 2012). The adhesion of cells to the ECM and the deformation of the ECM can regulate the activity of Yap, influencing the proliferation and apoptosis of chondrocytes through FA (Puleo et al. 2019). Additionally, the deletion of Piezo1 leads to Yap nuclear rejection during the development of human neural stem cells and the zebrafish outflow valve, indicating that Piezo1 can act upstream of Yap (Pathak et al. 2014; Duchemin et al. 2019). Certain studies have shown that Piezo1 is a target of Yap transcription, and that Yap can promote the expression of Piezo1 (Hasegawa et al. 2021). However, the relationship between Yap and Piezo1 in the process of IVDD remains unclear.

A number of risk factors can induce oxidative stress, including aging and mechanical stress. Oxidative stress response is often accompanied by the activation of NLRP3/caspase-1 inflammasome and NF—κ B signaling pathway, which can form an inflammatory response and lead to cell apoptosis, playing an important role in promoting the occurrence and development of IVDD (Li et al. 2022; Zhang et al. 2022a, b, c).

In the present study, a mouse model of spinal degeneration was established to explore the differences in Piezo1 expression between the normal and model groups. Additionally, chondrocytes were isolated and cultured to study the mechanism of apoptosis induced by Piezo1 protein under different conditions. The present study clarifies the role of Piezo1 protein in the progression of IVDD, provides evidence for the development of new therapeutic strategies and presents new insights for delaying the occurrence and development of IVDD.

## Materials and methods

### Reagents

Yoda1 (cat. no. HY-18723), GsMTx4 (cat. no. HY-P1410A) and Verteporfin (cat. no. HY-B0146) are purchased from MedChemExpress, Latrunculin A (cat. no. 10010630) was purchased from Cayman Chemical Company and BAPTA-AM (cat. no. S753401) was purchased from Selleck Chemicals.

### Cell isolation and culture

All animal experiments were approved by the Animal Ethics Committee of Shandong Provincial Hospital Affiliated to Shandong First Medical University (Jinan, China; approval number: 2023–129). Chondrocytes were isolated from 72 C57BL/6J mice aged 5–7 days. The sacrifice method for mice (5–7 days, 5 weeks old or 12 weeks old) was via cervical dislocation. When the breathing and heartbeat of mice stop or their pupils dilate and corneal reflexes disappear, we consider euthanasia successful. During dissection, a microscope was used to remove the muscles and ligaments around the spine and extract cartilage tissue from the vertebral growth plate. The tissues were then digested using 0.25% EDTA trypsin in an incubator at 37 ˚C with 5% CO_2_ for 30 min. After centrifugation at 440 xg for 10 min at room temperature, the supernatant was removed, and the pellet was digested in a type II collagenase mixture (1 g collagenase type II: 2 ml complete medium) for 1 h under the same incubator conditions. The mixture was then centrifuged at 200 xg for 10 min at room temperature and the supernatant was removed. Type II collagenase mixture was used for further digestion for 5 h and the supernatant was removed again. The cell suspension was prepared by precipitation and mixed with complete medium [10% FBS (ExCell Bio.; cat. no, FSP500), streptomycin sulfate (100 mg/ml), penicillin (100 U/ml) and DMEM/F12 (Shanghai BasalMedia Technologies Co., Ltd.; cat. no, L310KJ)]. The cell suspension was then transferred to a cell culture bottle and incubated at 37˚C with 5% CO_2_. First or second-generation chondrocytes were used in the experiments.

### Animal model development and treatment

This study used 24 healthy 5-week-old male C57BL/6J mice (Jinan Pengyue Experimental Animal Breeding Co., Ltd) with an initial average weight of 20g, which were randomly divided into four groups: The control, IVDD, IVDD + Yoda1 and IVDD + GsMTx4 groups. The mice were housed in the SPF level specialized feeding room of the animal laboratory at Shandong Provincial Hospital. Starting from the mice entering the laboratory, we conduct behavioral and health tests on mice every 2 days, including observing their food and water intake, measuring body temperature, examining their eyes, ears, hair, and skin, and checking their feces and urine. After one week of adaptation to the environment, mice were anesthetized with an appropriate amount of Avertin (14uL/g) intraperitoneally. Subsequently, the IVDD mouse model was established by cutting the bilateral facet joint as well as the supraspinous and interspinous ligament in the L4/5 sites (de Oliveira et al. 2018; Ao et al. 2019). Then, 1 week after surgery, the IVDD + Yoda1 group mice were injected with 4 µg/kg/day Yoda1 in 100 μl working solution [10% DMSO and 90% (20% SBE-β-CD in saline)] into the tail vein, and the IVDD + GsMTx4 group mice were injected with 0.4 µg/kg/day GsMTx4 in 100 μl working solution (10% DMSO, 40% PEG300, 5% Tween-80 and 45% saline) into the tail vein (Zhao et al. 2021). The mice in the control group were injected intravenously without the aforementioned drug solution (10% DMSO and 90% saline) every day. After 12 weeks, the mice were euthanized following the humane endpoint (as aforementioned) and the vertebral bodies of L3-L5 were collected and examined by micro-computed tomography (CT), histology and immunohistochemistry.

### Micro-CT analysis

The collected vertebral samples were fixed in 4% paraformaldehyde solution at room temperature for 24 h. The microstructures of the vertebral body and endplate were scanned by micro-CT (vivaCT 40) at a resolution of 10.5 μm, 100 kV and 98 μA. The internal program of the system was used to evaluate the intervertebral disc height and bone volume / tissue volume-related parameters.

### Histological staining and immunohistochemistry analysis

The collected vertebral samples were fixed in 4% paraformaldehyde solution at room temperature for 24 h, then decalcified in 10% EDTA solution at room temperature, with a 50X sample volume for 3 weeks. The decalcified solution was refreshed every 3 days. After vertebral softening, the paraffin-embedded sections were cut into 5 μm thick sagittal sections for hematoxylin–eosin staining [Hematoxylin Staining: Put sections into Hematoxylin solution at room temperature for 3–5 min, rinse with tap water. Eosin staining: Place the sections in 95% ethanol at room temperature for 1 min, eosin dye for 15 s (Wuhan Servicebio Technology Co., Ltd.; cat. no. G1076)] and immunohistochemical examination of the CEP. The severity of IVDD was assessed by three spinal surgeons, and the histological evaluation of the intervertebral disc is shown in Table 1. After dewaxing (Dewaxing hydration: Before dewaxing, bake the slices at 65 °C for 60 min, then soak the tissue slices in xylene for 7 min, replace the xylene for another 7 min, replace the xylene for another 7 min, soak in anhydrous ethanol for 5 min, replace the anhydrous ethanol for another 5 min, and then soak in 95% ethanol, 85% ethanol, 75% ethanol, and 50% ethanol for 5 min each. Finally, soak in ultrapure water for 5 min), antigen retrieval (Antigen retrieval: Circle the tissue with an immunohistochemical pen (Vector Laboratories.; cat. no. H-4000) add diluted proteinase K (Shandong Sparkjade Biotechnology Co., Ltd.; cat. no. AA1907) solution to the slice, and then repair it at 37 ℃ for 30 min in a moisturizing box. Then, add endogenous peroxidase blocking droplets (Shandong Sparkjade Biotechnology Co., Ltd.; cat. no. EE0007) onto the slices and block at room temperature for 10 min to remove endogenous peroxidase.) and block (Block: Add 10% BSA (Lanjieke Technology Co., Ltd.; cat. no. BS114-25g) dropwise to the slices and seal at 37 ℃ for 30 min, then shake off the blocking serum), the tissue sections were incubated overnight with type II collagen (COL2; Proteintech Group, Inc.; cat. no. 28459-1-AP; 1:400) MMP3 (Wuhan Servicebio Technology Co., Ltd.; cat. no. GB11131-50; 1:400) and Piezo1 (Proteintech Group, Inc.; cat. no. 15939‐1‐AP; 1:200) primary antibodies at 4 ˚C. Then, the tissues were incubated with the secondary antibody (BOSTER Biological Technology, Ltd.; cat. no. BA1056; 1:5,000) at room temperature for 15 min. An Olympus Optical microscope (Nikon Corporation of Japan) was used for observation, and cellSens 2017 software was used for analysis.

**Table 1:** Lumbar intervertebral disc degeneration assessment scoring system

Score	Nucleus pulposus	Annulus fibrosus	Osteophyte
0	Bulging gel with abundant notochordal cells	Compact fibrous lamellas	Absence
1	Notochordal cells loss; chondrocyte-like cells emergence	Proliferation of fibrocartilaginous tissue and loss of nuclear-annular border	Appearance
2	Focal mucoid degeneration; clefts	Fissures in annulus fibrosis	Overgrowth
3	Diffuse mucoid degeneration and clefts throughout nucleus		

### Annexin V-FITC/PI staining and flow cytometry

Chondrocytes were inoculated into a 6-well plate at a density of 1 × 10^5^ cells per well. When the cell density reached 70–80%, four groups are set up for intervention as follows: The control (No medication), TBHP (100 μM, 6 h), Yoda1 (30 μM, overnight) and GsMTx4 (5 μM, overnight) groups. After the intervention, the cells were collected by centrifugation at room temperature for 5 min at 200 xg and stained for 20–30 min in the dark using apoptosis detection kits (Cyagen Biosciences, Inc.) containing annexin V-FITC/PI. The apoptosis rate of chondrocytes was calculated by the BD FACSDiva 4.1 software (Becton, Dickinson and Company), as follows: number of early apoptotic cells (annexin V^+^ / PI^−^) + number of late apoptotic cells (annexin apoptotic PI^+^ / total cells).

### ROS assay

Chondrocytes were inoculated into a 6-well plate at a density of 1 × 10^5^ cells per well. The seeded plates were divided into six groups and when the cell density reached 70–80%, the wells are treated as follows: CTRL (No medication), TBHP (100 μM, 6 h), Yoda1 (30 μM, 24 h), TBHP + Yoda1, si-Piezo1 (100 nM, 72 h), or TBHP + si-Piezo1. A Reactive Oxygen Species Kit (Beyotime Institute of Biotechnology; cat. no. S0033) was then used to evaluate the ROS levels in the chondrocytes. The cells were washed with serum-free DMEM (Shanghai BasalMedia Technologies Co., Ltd.; cat. no, L110KJ) twice, and then incubated with 10 μM DCFH-DA in a 37 ˚C dark environment for 20 min. Finally, the cells were collected, and the average fluorescence intensity was measured by the BD LSRFortessa instrument and analyzed by the BD FACSDiva 4.1 software.

### Alkaline phosphatase (ALP) staining and activity assay

Chondrocytes were inoculated into a 12-well plate at a density of 1 × 10^5^ cells per well. When the density reached 70–80%, osteogenic differentiation medium (Cyagen Biosciences, Inc.) was added and incubated for 7 days. On the sixth day, drugs were added for intervention (as aforementioned). The cells were then stained with p-nitrophenyl phosphate using the Alkaline Phosphatase Detection Kit (Beyotime Institute of Biotechnology.; cat. no. P0321S). A mixture of 50 ml of cell lysate and 50 ml of buffer was incubated at 37 ˚C for 10 min, after which the termination buffer was added. The total protein concentration was determined by BCA protein assay kit (Beijing Solarbio Science & Technology Co., Ltd.; cat. no. PC0020). The ALP activity was measured by determining the optical density (OD) at 405 nm of the total protein content (per mg).

### Staining with alizarin red

Chondrocytes were inoculated into a 12-well plate at a density of 1 × 10^5^ cells per well. When the cells reached 70–80% confluency, osteogenic differentiation medium (Cyagen Biosciences, Inc.) was added. The following four intervention groups are set up after 20 days of culture: The control (No medication), TBHP (100 μM, 6 h), Yoda1 (30 μM, overnight) and GsMTx4 (5 μM, overnight) groups. On the 21st day, the cells were washed with PBS twice, fixed with 4% paraformaldehyde at room temperature for 30 min, and then incubated with alizarin red solution (Cyagen Biosciences, Inc.) at room temperature for 30 min. Quantitative methods were used to evaluate the mineralization level of the chondrocytes. After dissolving the alizarin red in 10% acetylpyridine (Sigma-Aldrich; Merck KGaA) solution at room temperature for 1 h, the OD of the alizarin red was measured at 570 nm using a spectrophotometer.

### siRNA transfection

Chondrocytes were inoculated into a 6-well plate at a density of 1 × 10^5^ cells per well and divided into the control (No medication), TBHP (100 μM, 6 h), si-Piezo1(100 nM, 72 h), TBHP + si-Piezo1, TBHP + Yoda1 (30 μM, overnight) and TBHP + Yoda1 + si-Yap (100 nM, 72 h) groups. The cells were cultured in a 37˚C and 5% CO_2_ incubator with DMEM containing 10% FBS. Transfection was conducted according to the groups when the cell density reached 70–80%. The ribo*FECT*^TM^CP reagent (Guangzhou RiboBio Co. Ltd.) was used in the transfection of siRNA targeting the mouse Piezo1 or Yap gene. The sequence of siRNAs were as follows: si-Yap 5'-UGAGAACAAUGACAACCAAUAdTdT-3'; si-Piezo1 5'-AGAAGAAGAUCGUCAAGUAdTdT-3' and negative control, 5'-UUCUCCGAACGUGUCACGUTTdTdT-3' (Research Cloud Biotechnology Co., Ltd). After transfection at 37 °C for 72 h, western blotting analysis was used to evaluate the effectiveness of Piezo1 and Yap protein knockdown.

### Western blotting analysis

Chondrocytes were inoculated into a 6-well plate at a density of 5 × 10^5^ cells per well. Intervention was conducted (as aforementioned) when the cell density reached 80%. After the intervention, the cells were washed with PBS and then 100 μl of RIPA lysis buffer containing 1% protease inhibitor mixture (BOSTER Biological Technology, Co., Ltd.; cat. no. AR0102) was added per well on the ice for 40 min. The lysate was collected and centrifuged at 15365 xg and 4 ˚C for 20 min. The supernatant was collected, and the protein concentration was measured using a BCA kit. The rest of the samples were mixed with fast-soluble protein buffer according to the volume, and the protein was denatured at 100˚C for 15 min. The mass of protein per lane was calculated based on the total protein content of different groups, and then the proteins were separated by electrophoresis using a 6–12% sodium dodecyl sulfate polyacrylamide gel and then transferred to a polyvinylidene fluoride membrane (MilliporeSigma). After transferer, the membrane was blocked with 5% skimmed milk powder at room temperature for 2 h, washed with TBST [Contains 0.1% Tween (Beijing Solarbio Science & Technology Co., Ltd.; cat. no. T8220)] three times and then incubated with the corresponding primary antibody at 4˚C overnight. Following primary antibody incubation, the membranes were washed with TBST three times and incubated with the corresponding secondary antibody (BOSTER Biological Technology, Ltd.; cat. no. BA1065 1:5,000) at room temperature for 2 h. The bands were developed by Western ECL substrate kit (Pierce; Thermo Fisher Scientific, Inc.) and analyzed by Bio-Rad scanner (Bio-Rad Laboratories, Inc.). The bands were quantified by ImageJ 2023 (National Institutes of Health). The following primary antibodies were used in this experiment: Piezo1 (Proteintech Group, Inc.; cat. no. 15939‐1‐AP; 1:2,000), MMP3 (BOSTER Biological Technology, Ltd.; cat. no. BM4074; 1:500), MMP13 (Proteintech Group, Inc.; cat. no. 18165-1-AP; 1:1,000), SOX9 (Proteintech Group, Inc.; cat. no. 18165-1-AP; 1:1,000), COL2 (Proteintech Group, Inc.; cat. no. 28459-1-AP; 1:2,000), IκB (ABclonal Biotech Co., Ltd; cat. no. A23223; 1:500), phosphorylated (p-)IκB (ABclonal Biotech Co., Ltd; cat. no. AP0707; 1:500), p65 (ABclonal Biotech Co., Ltd.; cat. no. AP0475; 1:500), p-p65 (ABclonal Biotech Co., Ltd.; cat. no. A2547; 1:500), Cytochrome c (Proteintech Group, Inc.; cat. no. 10993-1-AP; 1:1,000), cleaved-Caspase3 (Cohesion Biosciences, Inc.; cat. no. CPA1137; 1:2,000), BAX (Proteintech Group, Inc.; cat. no. 60267‐1‐Ig; 1:2,000), BCL2 (Cohesion; cat. no. CPA1095; 1:2,000), RUNX family transcription factor 2 (RUNX2; CST Biological Reagents Co., Ltd.; cat. no. 12556; 1:1,000), COL10 (BOSTER Biological Technology, Ltd.; cat. no. BA2023; 1:500), Yap (Proteintech Group, Inc.; cat. no. 13584-1-AP; 1:2,000), p-Yap (Proteintech Group, Inc.; cat. no. 29018-1-AP; 1:2,000), connective tissue growth factor (CTGF; Proteintech Group, Inc.; cat. no. 25474-1-P; 1:1,000), NLRP3 (Proteintech Group, Inc.; cat. no. 68102-1-Ig; 1:2,000), Caspase-1 (Proteintech Group, Inc.; cat. no. 22915-1-AP; 1:2,000), β‐actin (Proteintech Group, Inc.; cat. no. 81115‐1‐RR; 1:2,000) and GAPDH (Proteintech Group, Inc.; cat. no. 10494-1-AP; 1:1,000).

### Determination of intracellular Ca^2+^ content

The intracellular Ca^2+^ content was measured using pCMV-jGCaMP7c (Beyotime Institute of Biotechnology.; cat. no. D2863). Chondrocytes were inoculated into a 12-well plate at a density of 2–7 × 10^5^ cells per well. Drug intervention was then applied to the cells as aforementioned. After the intervention, the medium was replaced with 2 ml of fresh culture medium per well and transfection was conducted. For transfection, 50 μl of DMEM without antibiotics and serum was added, followed by 1 μg plasmid DNA. Then 1.6 μl Lipo8000™ transfection reagent (Beyotime Institute of Biotechnology; cat. no. C0533) was added and gently mixed, avoiding vortexing or centrifugation. After 24–48 h of incubation in the incubator, successful transfection effect was observed using a fluorescence microscope.

### Determination of cytoskeleton morphology

The cytoskeleton was examined using Actin-Tracker Red-594 (Beyotime Institute of Biotechnology; cat. no. C2205S). Chondrocytes were inoculated into a 12-well plate at a density of 5 × 10^5^ cells per well. After the intervention (as aforementioned), the cells were fixed with immunostaining fixative (Beyotime Institute of Biotechnology.; cat. no. P0098) at room temperature for 10–20 min and washed 2–4 times with immunostaining wash solution (Beyotime Institute of Biotechnology; cat. no. P0106) for 5 min each time. Then, immunofluorescence staining secondary antibody dilution solution (Beyotime Institute of Biotechnology; cat. no. P0108) was used to dilute Actin Tracker Red to prepare the working solution. 1mL of working solution was added to each well and incubate at room temperature in the dark for 30–60 min. Finally, the cells were incubated with 4,6-diamino-2-phenylindole (DAPI) for at room temperature 10 min. The detergent was washed for three times and the morphology of the cytoskeleton was observed using a fluorescence microscope.

### Immunofluorescence staining

Chondrocytes were inoculated into a 24-well plate at a density of 5 × 10^5^ cells per well. When the cells reached 70% confluency, the intervention was conducted according to the corresponding groups (as aforementioned). After the intervention, the cells were fixed with 4% paraformaldehyde at room temperature for 20 min and washed with PBS three times. Then, the cells were incubated with 0.5% Triton X-100 at room temperature for 20 min, washed with PBS three times, blocked with 5% BSA for 1 h at room temperature and washed again with PBS three times. The cells were then incubated overnight with antibodies against Piezo1 (Proteintech Group, Inc.; cat. no. 15939-1-AP; 1:200), Yap (Proteintech Group, Inc.; cat. no. 13584-1-AP; 1:200) and COL2 (Proteintech Group, Inc.; cat. no. 28459-1; 1:200) at 4 ˚C. Next, the cells were incubated with goat anti-rabbit secondary antibody conjugated with Cy3 (Beyotime Institute of Biotechnology.; cat. no. A0516; 1:250) at room temperature in the dark for 1 h. After washing with PBS three times and incubation with DAPI at room temperature for 5 min, the fluorescence intensity was observed by a fluorescence microscope (Evos FL Auto; Thermo Fisher Scientific, Inc.).

### Statistical analysis

Adobe Photoshop 2023 (Adobe Systems, Inc.) was used to cut the protein bands, ImageJ 2023 (National Institutes of Health) was used to quantify the protein density and GraphPad Prism 10 (Dotmatics) was used for data analysis. The unpaired Student’s t-test was used for comparing two groups and one-way ANOVA followed by the Tukey test were used for multiple comparisons. The bar graphs represent the mean ± SD of cumulative results from independent experiments. P < 0.05 were considered to indicate a statistically significant difference.

## Results

### Piezo1 is involved in the process of IVDD

**Fig. 1:**
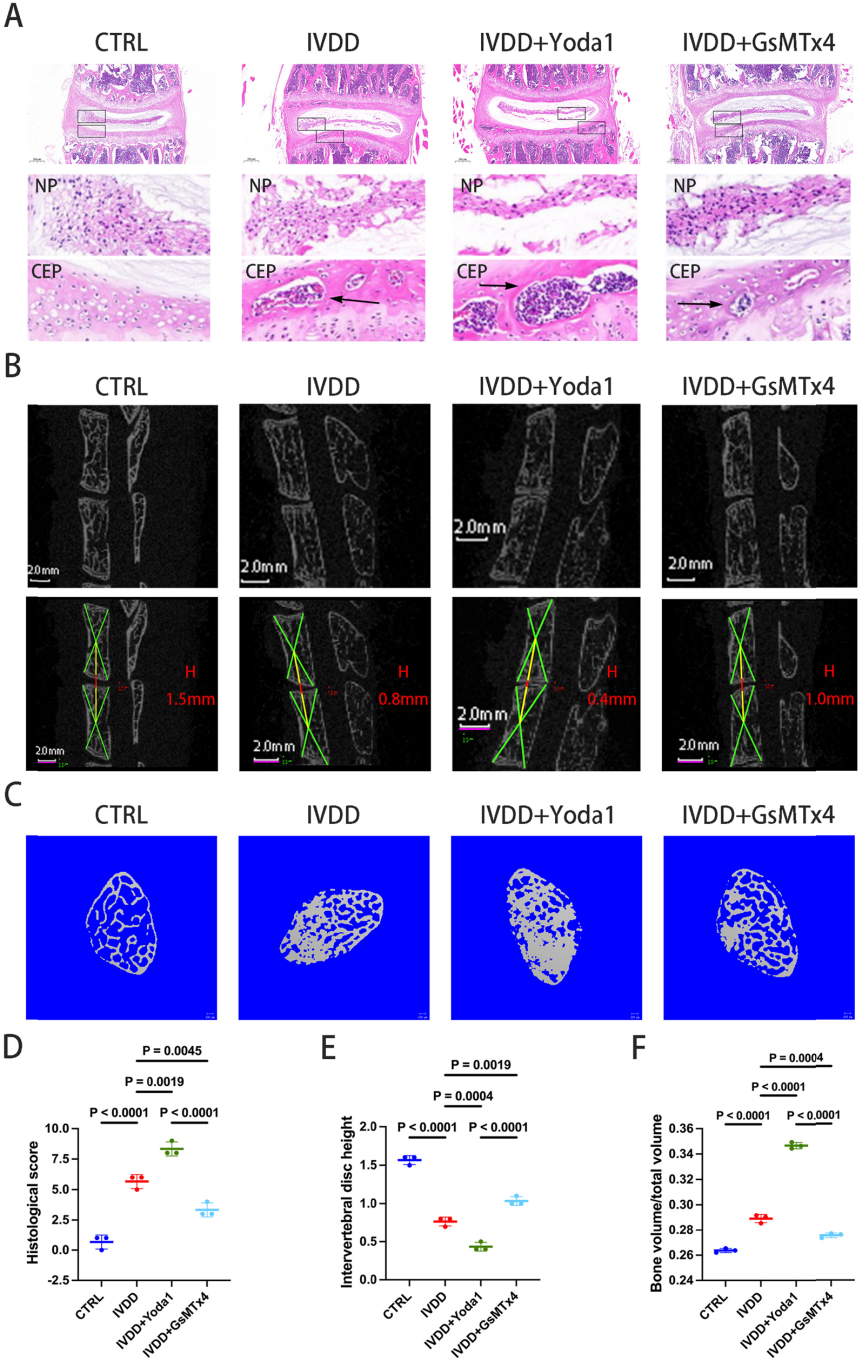
Piezo1 is involved in intervertebral disc degeneration. **A** The L4/5 level intervertebral discs from the CTRL, IVDD, IVDD + Yoda1 and IVDD + GsMTx4 groups were stained with hematoxylin–eosin. The black arrow highlights the bone tissue (scale bar, 200 μm). **B** The height of the intervertebral discs in the four groups were analyzed by Micro-CT (scale bar, 2.0 mm). **C** The CEPs from the four groups were analyzed using Micro-CT to detect the bone density (scale bar, 100 μm). **D** Analysis of the histological scores of the lumbar vertebrae from the four groups. **E** Histomorphometry analysis of the intervertebral disc heights of the four groups. **F** Bone volume/total volume was used to evaluate the calcification in the CEP. Data are presented as the mean ± SD. *CEP* cartilage endplate, *NP* nucleus pulposus, *Micro-CT* micro-computed tomography, *CTRL* control, *IVDD* intervertebral disc degeneration

Following the establishment of a spinal degeneration model, more mineralized bone tissue in the CEP layer of mice was observed in the IVDD group compared with the control group, with a significant increase in the histological score of the intervertebral disc (Table 1 and Fig. 1D). Based on these results, the spinal degeneration model was re-established and the IVDD mice were injected with either the Piezo1 agonist, Yoda1, or the inhibitor, GsMTx4. Compared with the IVDD group, the calcification of CEP and the deformation of AF and NP were significantly increased in mice injected with agonists (Fig. 1A). Conversely, in the mice injected with the inhibitor, the CEP exhibited a higher amount of gelatinous NP tissue and a lower amount of bone tissue (Fig. 1A), along with an decreased histological score of the intervertebral discs (Fig. 1D). The results indicated that Yoda1 accelerated the degeneration of the intervertebral disc, whereas GsMTx4 slowed it down.

Three-dimensional imaging and the bone mineral density (BMD) of the vertebral specimens from the mice were measured using micro-CT. A comparison between the groups revealed a significant decrease in intervertebral disc height (Fig. 1B and E) and a significant increase in the BMD of the CEP (Fig. 1C and F) in the IVDD group compared with the control group. After injection with Yoda1, the height of the intervertebral disc further decreased and the BMD increased, while injection with GsMTx4, resulted in an increase in the intervertebral disc height and a decrease in the BMD (Fig. 1B, C, E and F). The results suggested that regulating the expression of Piezo1 protein can affect the progress of IVDD and the denaturation of the CEP.

### Piezo1 contributes to oxidative stress-induced CEP degeneration

**Fig. 2:**
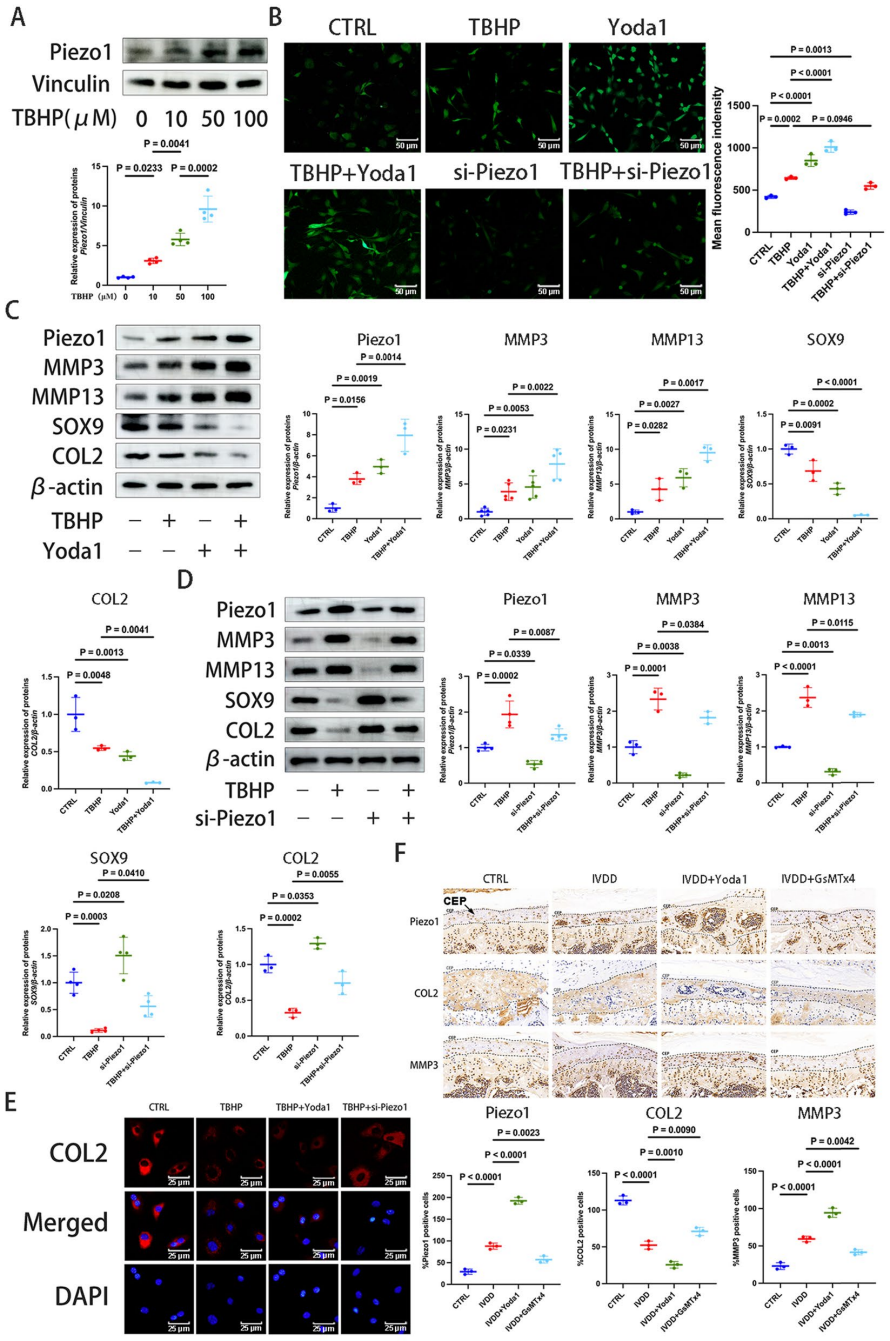
Piezo1 contributes to oxidative stress-induced CEP degeneration. **A** Detection of Piezo1 protein expression in chondrocytes by western blotting following intervention with a TBHP concentration gradient (0, 10, 50 and 100 μM) for 6 h, which was semi-quantified by ImageJ. **B** Intracellular ROS levels in chondrocytes was detected by immunofluorescence (scale bar, 50 μm). **C**, **D** Western blotting was used to detect the protein expression levels of Piezo1, MMP3, MMP13, SOX9 and COL2 in chondrocytes pretreated with Yoda1 (30 μM) for 24 h, si-Piezo1 (100 nM) for 72 h, and then TBHP (100 μM) for 6 h. The protein band density was then calculated. **E** Immunofluorescence staining was performed to evaluate the presence of COL2 (red) expression in chondrocytes pretreated with Yoda1 (30 μM) for 12 h or si-Piezo1 (100 nM) for 72 h and then TBHP (100 μM) for 6 h (scale, 25 μm). **F** Immunohistochemical staining for Piezo1, COL2 and MMP3 was performed on tissues from intervertebral disc degeneration model mice. Using a microscope at a magnification of × 400, 3 slices were collected from 3 mice to quantify the ratio of Piezo1, COL2 and MMP3 positive cells. Data are presented as the mean ± SD. *CTRL* control; si-Piezo1 Piezo1 small interfering (si)RNA, *CEP* cartilage endplate, *ROS* reactive oxygen species, *TBHP* tert-Butyl hydroperoxide, *COL2* type II collagen, *IVDD* intervertebral disc degeneration

TBHP was used to simulate the process of IVDD in chondrocytes. As the TBHP concentration increased (0, 10, 50 and 100 μM), the expression of Piezo1 protein gradually increased in a dose-dependent manner (Fig. 2A). Yoda1 is a selective Piezo1 agonist that can enhance Ca^2+^ influx, while si-Piezo1 is a small interfering (si)RNA that selectively knockout the Piezo1 gene to inhibit Ca^2+^influx. To explore the relationship between Piezo1 protein and oxidative stress, immunofluorescence experiments were conducted, and it was found that TBHP exposure led to a significant increase in ROS. Following Yoda1 intervention, both Piezo1 and the ROS level significantly increased, while si-Piezo1 intervention reduced both (Fig. 2B, C and D). The results indicated that Piezo1 expression can be promoted or inhibited, which can increase or decrease the level of oxidative stress in chondrocytes.

Oxidative stress can lead to degradation of the chondrocyte matrix (Wang et al. 2022a, b). Western blotting analysis showed that Yoda1 increased the MMP3 and MMP13 levels, while reducing the SOX9 and COL2 levels (Fig. 2C). Conversely, si-Piezo1 reduced Piezo1, MMP3 and MMP13 expression, while increasing the SOX9 and COL2 levels (Fig. 2D). Additional immunofluorescence results showed that regulating Piezo1 expression with Yoda1 and si-Piezo1 affected COL2 expression (Fig. 2E). These findings indicated that modulating Piezo1 protein expression can accelerate or slow ECM degradation, thereby regulating IVDD.

Immunohistochemical analysis of the tissues from the spinal degeneration model confirmed that, compared with the control, the IVDD group mice exhibited increased Piezo1 and MMP3 expression in the CEP layer, decreased COL2 expression and enhanced IVDD (Fig. 2F). Injecting Yoda1 into the IVDD mice promoted Piezo1 expression, increased IVDD, upregulated MMP3 and downregulated COL2 expression. Conversely, GsMTx4 injections inhibited Piezo1, delayed degeneration and increased COL2 expression (Fig. 2F).

### Piezo1 promotes CEP chondrocyte apoptosis and calcification

**Fig. 3:**
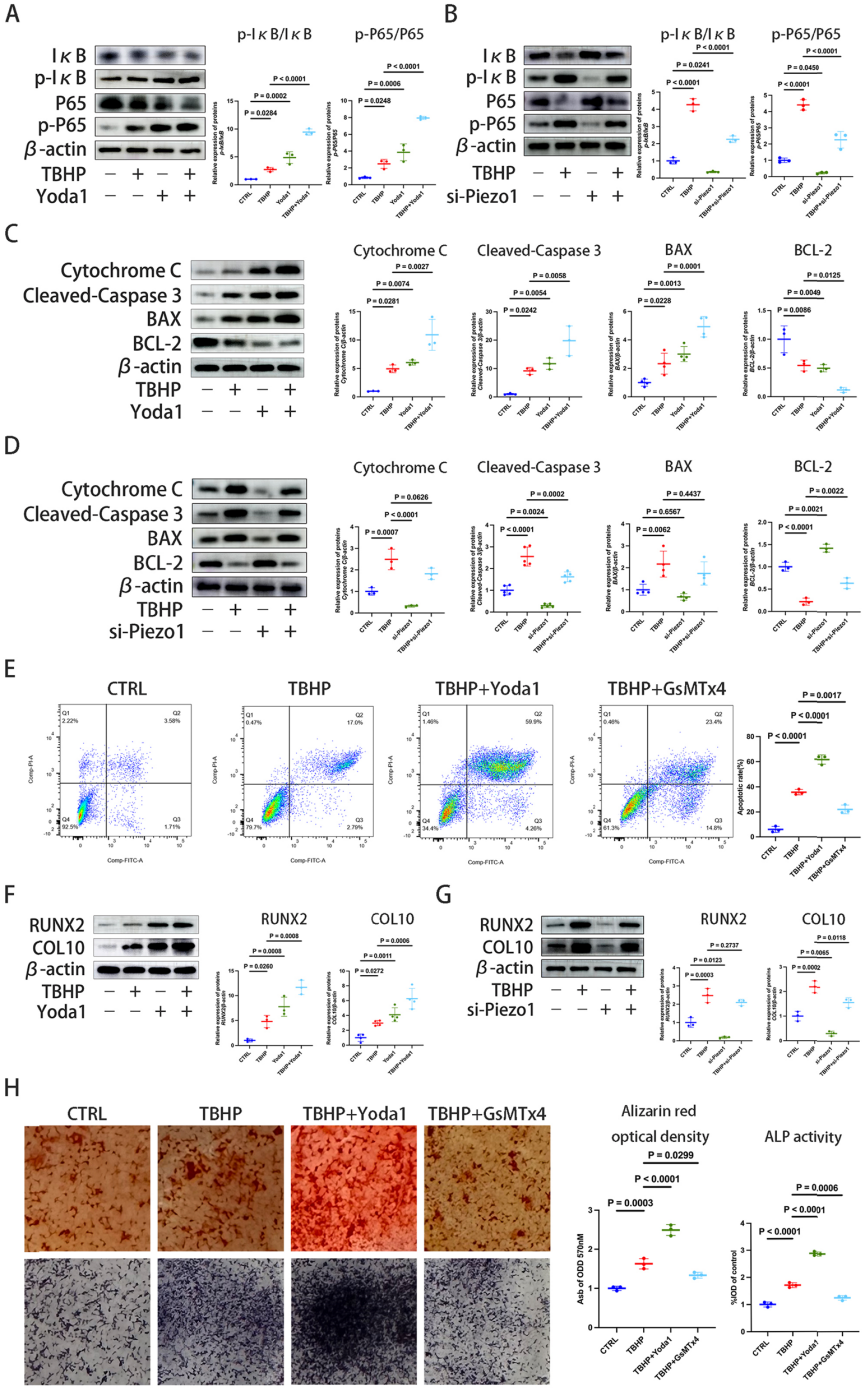
Piezo1 promotes cartilage endplate chondrocyte apoptosis and calcification. **A**, **B** Western blotting was used to detect the p-IκB/IκB and p-p65/p65 protein levels following intervention with the Piezo1 agonist, Yoda1 (30 μM) for 24 h, the Piezo1 inhibitor, si-Piezo1 (100 nM) for 72 h, and TBHP (100 μM) for 6 h, which were then semi-quantified using ImageJ. **C**, **D** Western blotting was used to measure the expression levels of the apoptosis-related proteins, Cytochrome c, cleaved-Caspase 3, BAX and BCL-2 after stimulating or inhibiting the expression of Piezo1, which were then semi-quantified using ImageJ. **E** Chondrocytes were treated with Yoda 1 or GsMTx4 followed by TBHP intervention. The cell apoptosis rates between groups were then compared following staining with Annexin V-FITC/PI and analysis using flow cytometry. **F**, **G** Western blotting was used to detect the osteogenic differentiation markers, RUNX2 and COL10, in chondrocytes following treatment with Yoda 1 or si-Piezo1 and intervention with TBHP. **H** Alizarin red and ALP staining of endplate chondrocytes, and semi quantitative analysis of the mineralization of nodules in endplate chondrocytes and ALP activity. Data are presented as the mean ± SD. *CTRL* control, *si-Piezo1* Piezo1 small interfering (si)RNA, *TBHP* tert-Butyl hydroperoxide, COL10, type X collagen, p-, phosphorylated, *ALP* alkaline phosphatase, *RUNX2* RUNX family transcription factor 2

ROS produced by TBHP can increase the inflammatory response of chondrocytes, and the NF-κB pathway has an important role in inflammation and apoptosis (Li et al. 2022). Next, it was investigated whether regulating the expression of Piezo1 could affect the NF-κB pathway. As shown in Fig. 3A, TBHP treatment activated the NF-κB pathway, as indicated by the increased ratio of p-IκB/IκB and p-p65/p65. To further explore the relationship between Piezo1 and NF-κB, Piezo1 expression was enhanced following TBHP and Yoda1 treatment and a further increase in the p-IκB/IκB and p-p65/p65 ratios was observed (Figs. 2C and 3A). Conversely, inhibiting Piezo1 expression produced the opposite effect on these inflammatory markers (Figs. 2D and 3B). The results showed that Piezo1 was involved in the activation of the NF-κB pathway.

Chondrocytes, the only cell type in the CEP, exhibit increased apoptosis when Piezo1 is upregulated and reduced apoptosis when Piezo1 is inhibited (Chen et al. 2023). The western blot experiments in the present study yielded similar results. Under TBHP intervention, Piezo1 protein expression increased alongside Cytochrome C, cleaved-Caspase 3 and the pro-apoptotic protein, BAX, while expression of the anti-apoptotic protein, BCL2 decreased (Figs. 2C and 3C). Conversely, reducing Piezo1 expression resulted in the opposite changes in Cytochrome C, cleaved-Caspase 3, BAX and BCL2 levels (Figs. 2D and 3D). To further explore the relationship between Piezo1 and chondrocyte apoptosis, Annexin V-FITC/PI staining was used to evaluate the apoptosis rate of chondrocytes by flow cytometry. It was found that TBHP increased the apoptosis of chondrocytes, which was further exacerbated by Yoda1 but mitigated by GsMTx4 (Fig. 3E). In summary, promoting Piezo1 increased ROS production, activating the NF-κB pathway, leading to an inflammatory response and enhanced chondrocytes apoptosis; however, inhibiting the expression of Piezo1 had the opposite result, reducing inflammation and apoptosis.

Chondrocyte hypertrophy and osteogenic differentiation are induced by oxidative stress. CEP degeneration and calcification can prevent the vertebral body from supplying oxygen and nutrients to the intervertebral disc, thus promoting the formation of IVDD (Yuan et al. 2019). Therefore, the effect of Piezo1 on the ossification of the chondrocyte was studied. The western blot results revealed that Piezo1 inhibition reversed the effect of the oxidative stress-induced increase in the expression of COL10 and RUNX2 (Fig. 3F and G). Consistent with the aforementioned results, oxidative stress enhanced the formation of mineralized deposits in chondrocytes and enhanced the activity of ALP (Fig. 3H). Promoting the expression of Piezo1 further increased the formation of mineralized deposits and the activity of ALP, while inhibiting the expression of Piezo1 had the opposite result (Fig. 3H). In summary, Piezo1 expression can affect the ossification of chondrocytes.

### Piezo1 activation can promote Yap activation via Ca^2+^ influx and cytoskeleton polymerization

**Fig. 4:**
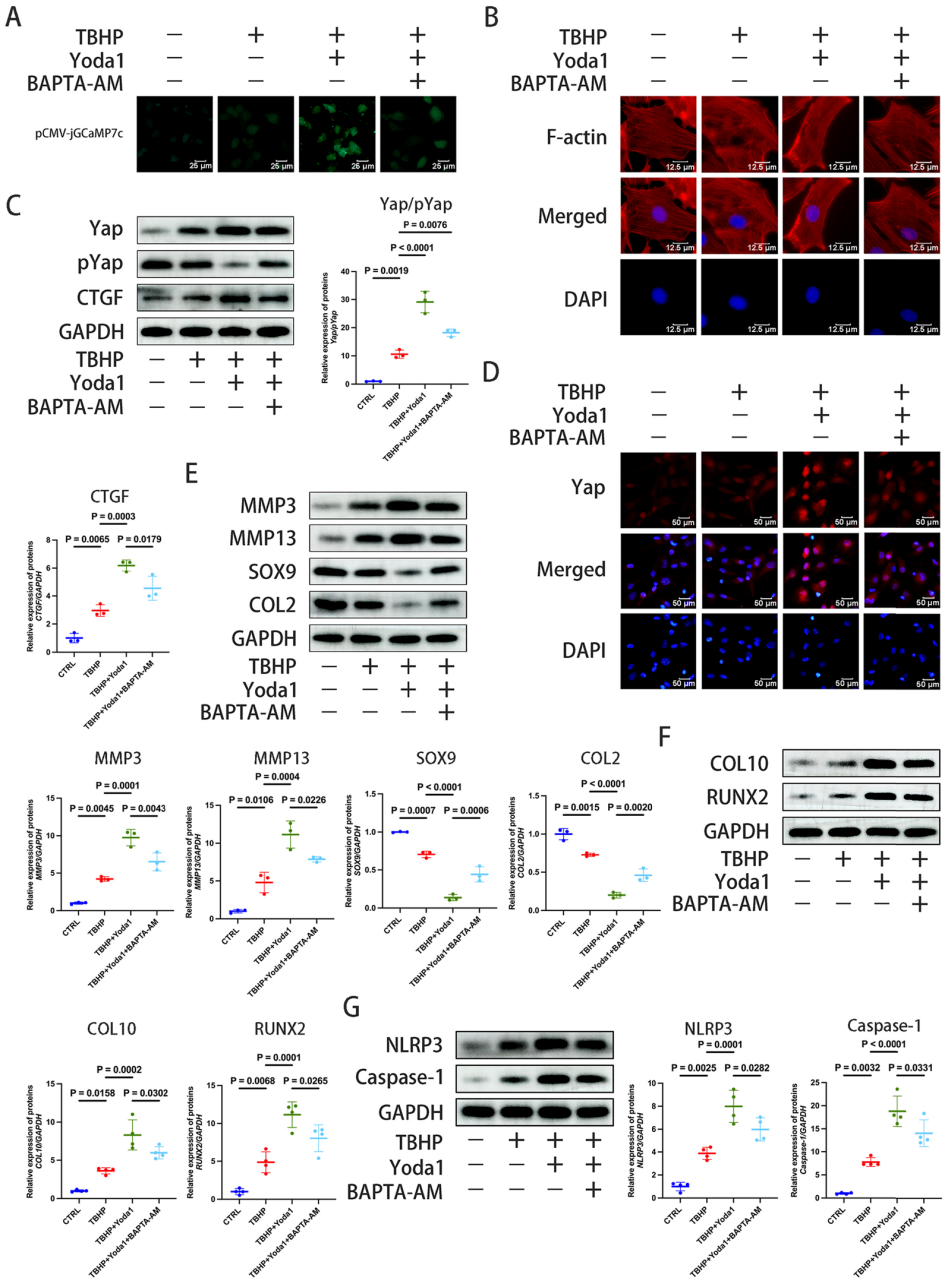
Piezo1 activation can promote Yap activation via calcium influx. **A** The intracellular calcium content in chondrocytes was determined following intervention with the Piezo1 agonist, Yoda1 (30 μM), for 24 h, TBHP (100 μM) for 6 h, and BAPTA-AM (5 μM) for 24 h using pCMV-jGCaMP7c. The intracellular calcium content was observed by the green fluorescence intensity using an inverted microscope (scale bar, 25 μm). **B** The cytoskeleton morphology was measured by Actin-Tracker Red-594 following intervention with Yoda1, TBHP and BAPTA-AM. The cytoskeleton morphology was observed under an inverted microscope (scale bar, 12.5 μm). **C** The protein levels of CTGF and the ratio of Yap/p-Yap were detected by western blotting analysis after intervention with Yoda1, TBHP and BAPTA-AM, which were semi-quantified by ImageJ. **D**, Immunofluorescence staining was performed to evaluate the Yap expression after intervention with BAPTA-AM (scale bar, 50 μm). **E**, **F**, **G**, Western blotting was used to detect the extracellular matrix degradation-related proteins, MMP3, MMP13, SOX9 and COL2, the osteogenic differentiation markers, COL10 and RUNX2, and the inflammatory response-related proteins, NLRP3 and Caspase-1, after intervention with Yoda1, TBHP and BAPTA-AM, which were semi-quantified by ImageJ. Data are presented as the mean ± SD. *CTRL* control; *TBHP* tert-Butyl hydroperoxide, *COL2/10* type II/X collagen, *p*- phosphorylated; *RUNX2* RUNX family transcription factor 2, *Yap* Yes-associated protein, *CTGF* connective tissue growth factor, *NLRP3* NLR family pyrin domain containing 3

Piezoelectric sensitive channels represent a class of cation-permeable channels that facilitate the influx of calcium ions (Ca^2^⁺) into cells. BAPTA-AM is a Ca^2+^ channel blocker that significantly inhibits Ca^2+^ influx in chondrocytes (Jing et al. 2021). Using the pCMV-jGCaMP7c probe, a significant decrease in Ca^2+^ influx in chondrocytes following BAPTA-AM treatment was detected (Fig. 4A). Immunofluorescence detection also showed restricted myofilament polymerization, which inhibited cytoskeletal polymerization (Fig. 4B). When the polymerization of the cytoskeleton is limited, the RhoA-mediated cytoskeletal morphology is changed (Xu et al. 2012), leading to a decreased ratio of Yap/p-Yap and CTGF expression, as shown in Fig. 4C. This result was confirmed by immunofluorescence (Fig. 4D). Additionally, expression of the ECM degradation-related proteins, MMP3 and MMP13, decreased, while SOX9 and COL2 expression increased, the chondrocyte ossification-related proteins, COL10 and RUNX2, decreased and the cellular inflammatory response level decreased, as indicated by the reduced levels of NLRP3 and Caspase-1 (Fig. 4E–G).

**Fig. 5:**
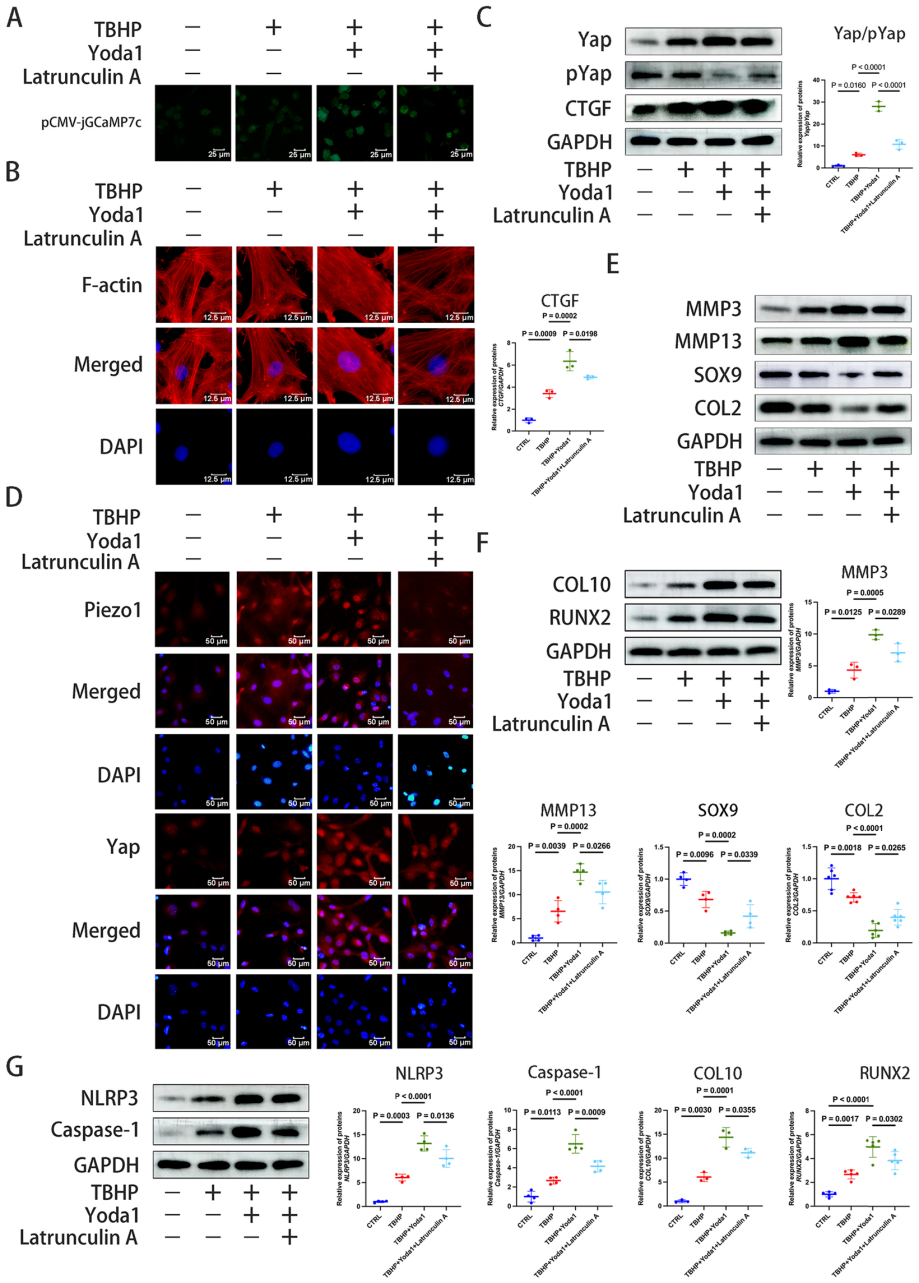
Piezo1 activation can promote Yap activation via cytoskeleton polymerization. **A** The intracellular calcium content in chondrocytes was determined following intervention with the Piezo1 agonist, Yoda1 (30 μM), for 24 h, TBHP (100 μM) for 6 h, and Latrunculin A (0.5 μM) for 30 min using pCMV-jGCaMP7c. The intracellular calcium content was observed by the green fluorescence intensity using an inverted microscope (scale bar, 25 μm). **B** The cytoskeleton morphology was measured by Actin-Tracker Red-594 following intervention with Yoda1, TBHP and Latrunculin A. The cytoskeleton morphology was observed under an inverted microscope (scale bar, 12.5 μm). **C** The ratio of Yap/p-Yap and the protein level of CTGF were detected by western blotting analysis after intervention with Yoda1, TBHP and Latrunculin, which were semi-quantified by ImageJ. **D** Immunofluorescence staining was performed to evaluate Piezo1 and Yap expression after treatment with Latrunculin A (scale bar, 50 μm). **E**, **F**, **G** Western blotting was used to detect the extracellular matrix degradation related-proteins MMP3, MMP13, SOX9 and COL2, the osteogenic differentiation markers, COL10 and RUNX2, and the inflammatory response-related proteins, NLRP3 and Caspase-1, after intervention with Yoda1, TBHP and Latrunculin A, which were semi-quantified by ImageJ. Data are presented as the mean ± SD. *CTRL* control; *TBHP* tert-Butyl hydroperoxide; *COL2/10* type II/X collagen, *p*- phosphorylated, *RUNX2* RUNX family transcription factor 2, *Yap* Yes-associated protein, *CTGF* connective tissue growth factor, *NLRP3* NLR family pyrin domain containing 3

Cytoskeleton polymerization plays a notable role in Piezo1-mediated signal transduction (Morachevskaya et al. 2021). The effects of the cytoskeleton inhibitor, Latrunculin A, on chondrocytes were next investigated in the present study. Latrunculin A not only reduced the influx of Ca^2+^, but also altered the morphology of the cytoskeleton and inhibited its aggregation (Fig. 5A and B). Western blotting analysis showed that inhibiting cytoskeleton polymerization decreased the Yap/p-Yap ratio and CTGF expression (Fig. 5C). Immunofluorescence also confirmed the decreased Yap and Piezo1 expression (Fig. 5D). Following the inhibition of cytoskeleton aggregation, the levels of the ECM degradation-related proteins, MMP3 and MMP13, decreased, SOX9 and COL2 expression increased, the cellular ossification levels decreased, as indicated by the reduced COL10 and RUNX2 expression, and the inflammatory response was downregulated, as indicated by the decreased NLRP3 and Caspase-1 levels (Fig. 5E, F and G). The results indicated that reducing calcium influx can regulate cytoskeleton morphology, restrict polymerization, decrease Yap activation, and reduce ROS production, thus slowing down the degradation of the ECM and the degeneration of the intervertebral disc.

### Yap downregulation can delay Piezo1-mediated chondrocyte degeneration and ECM degradation

**Fig. 6:**
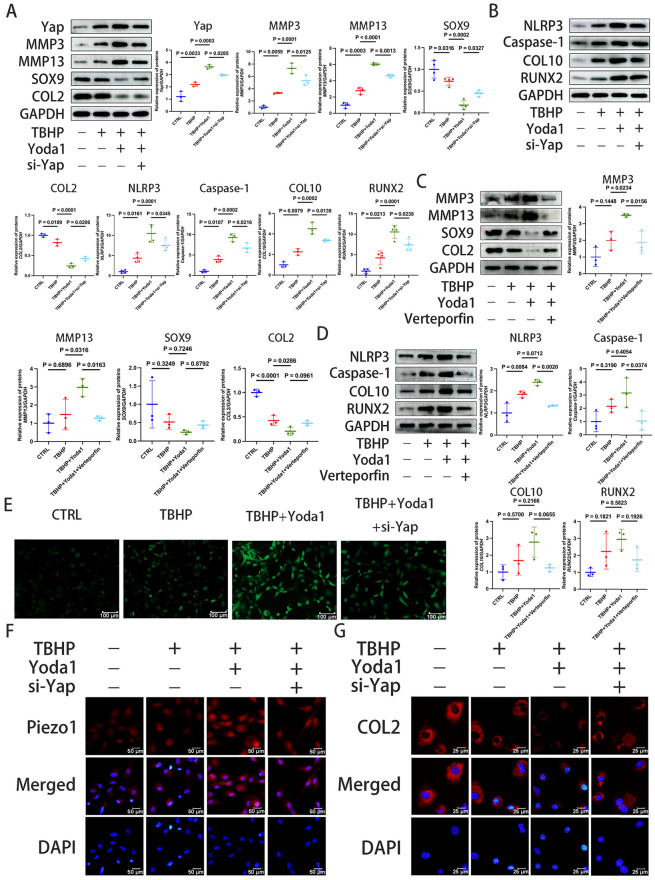
Yap inhibition can delay Piezo1-mediated chondrocyte degeneration and ECM degradation. **A**, **B** Western blotting analysis to determine levels of proteins, Yap, MMP3, MMP13, SOX9, COL2, COL10, RUNX2, NLRP3 and Caspase-1 in chondrocytes transfected with si-Yap (100 nM) for 72 h and then treated with TBHP (100 μM) for 6 h and Yoda1 (30 μM) for 24 h. **C**, **D** Western blotting analysis to determine levels of proteins, MMP3, MMP13, SOX9, COL2, COL10, RUNX2, NLRP3 and Caspase-1 in chondrocytes transfected with Verteporfin (0.2 μM) for 24 h and then treated with TBHP (100 μM) for 6 h and Yoda1 (30 μM) for 24 h. **E** Fluorescence images of the reactive oxygen species levels in chondrocytes (scale bar, 100 μm). **F** The expression of Piezo1 (red) in chondrocytes transfected with si-Yap was evaluated by immunofluorescence staining (scale bar, 50 μm). **G** The expression of COL2 in chondrocytes transfected with si-Yap was detected by immunofluorescence staining (scale bar, 25 μm). Data are presented as the mean ± SD. *CTRL* control, *TBHP* tert-Butyl hydroperoxide, *si-Yap* Yes-associated protein (Yap) small interfering (si)RNA, *COL2/10* type II/X collagen, *RUNX2* RUNX family transcription factor 2, *NLRP3* NLR family pyrin domain containing 3

**Fig. 7:**
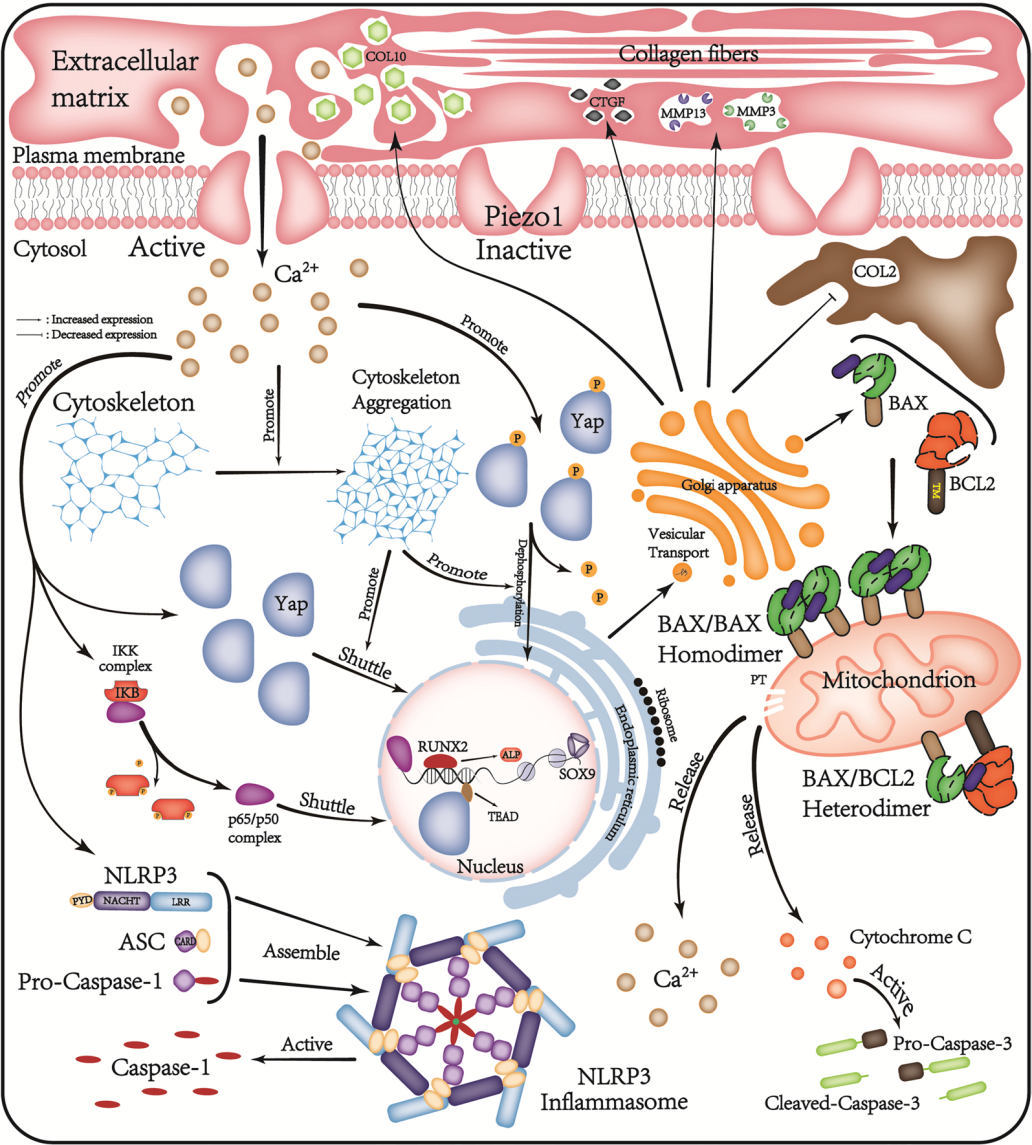
Piezo1/Ca^2+^/F-actin/Yap signaling axis. *Yap* Yes-associated protein, *CTGF* connective tissue growth factor, *COL2/10* type II/X collagen, *NLRP3* NLR family pyrin domain containing 3, *RUNX2* RUNX family transcription factor 2, *CaM* calmodulin, *ASC* apoptosis-associated speck-like protein containing a CARD, *ALP* alkaline phosphatase

Yap has an important role in mechanical stress-induced oxidative stress and apoptosis (Dupont et al. 2011). Piezo1 is the target of Yap signal transcription (Puleo et al. 2019; Hasegawa et al. 2021). To analyze the relationship between the Yap and Piezo1, si-Yap was synthesized and transfected into chondrocytes. Knocking down the Yap gene resulted in the decreased expression of Yap and restricted Piezo1 expression (Fig. 6F). The immunofluorescence results showed a decrease in ROS levels (Fig. 6E). Western blotting confirmed that promoting the expression of Piezo1 could accelerate ECM degradation; however, compared with the TBHP + Yoda1 group, increasing siRNA intervention to knock out Yap gene resulted in decreased expression of MMP3 and MMP13, and increased expression of SOX9 and COL2 (Fig. 6A). An immunofluorescence assay also confirmed the increase in COL2 expression (Fig. 6G). Higher oxidative stress levels are associated with increased cellular inflammatory responses (Zhang et al. 2022a, b, c). TBHP intervention elevated the expression of NLPR3 and its downstream Caspase-1, and promoting Piezo1 expression further increased the levels of NLRP3 inflammasomes (Fig. 6B). However, compared with the TBHP + Yoda1 group, increasing siRNA intervention to knock out Yap gene lowered the inflammatory response, as indicated by the decreased the NLRP3 and Caspase-1 levels, and reduced the levels of the markers of chondrocyte degeneration, COL10 and RUNX2 (Fig. 6B). Yap usually works when combined with TEAD. To further explore whether this mechanism exists, experiments were conducted using the Yap inhibitor, Verteporfin. The results showed that compared to the co intervention of TBHP and Yoda1, the expression of MMP3 and MMP13 decreased, the expression of SOX9 and COL2 increased, the expression of NLRP3 and Caspase-1 decreased, and the expression of COL10 and RUNX2 decreased (Fig. 6C, D). Verteporfin showed similar effects to si-Yap, indicating that Yap requires TEAD to exert its effects. In summary, the results indicated that Yap/TEAD is a downstream effector of Piezo1 and mediates its effect in chondrocyte degeneration and ECM degradation (Fig. 7).

## Discussion

IVDD is caused by a variety of factors, including heredity factors, an unhealthy lifestyle and aging (Kim et al. 2011; Cheung et al. 2010). As age increases, the severity of IVDD gradually increases, potentially leading to lower back pain or lower limb symptoms. Neglecting these symptoms can result in more serious spinal diseases, including disc herniation, spinal canal stenosis and scoliosis (Khan et al. 2017). Current treatment primarily involves medications that address symptoms but do not halt the progression of IVDD, highlighting the need to investigate the underlying causes of this condition. Inflammation, calcification, oxidation and other pathological states of the NP, AF and CEP can lead to IVDD (Xia et al. 2024). Studying their mechanisms of occurrence may assist in delaying or improving IVDD. At present, there have been several studies on inflammatory NP cells in IVDD (Zhang et al. 2022a, b, c; Lu et al. 2023; Sun et al. 2020). However, as the gateway for delivering nutrients to the intervertebral disc, the CEP plays a notable role, yet there has been limited research on its inflammation. Therefore, it is particularly important to study the inflammatory response mechanism of chondrocytes.

In vivo and in vitro studies have shown that endplate degeneration has an important role in the initiation and progression of IVDD (Zhong et al. 2016; Ding et al. 2014). Piezo1 protein is expressed in human mesenchymal stem cells, osteoblasts, osteoclasts and chondrocytes (Zhu et al. 2021). A study has shown that Piezo1 is also highly expressed in mouse chondrocytes (Lee et al. 2014). Since chondrocytes are the only cells in the CEP layer and their proliferation and apoptosis are crucial in the process of IVDD, understanding the role of Piezo1 in these cells could provide new insights into the mechanisms driving IVDD and identify potential therapeutic targets.

In the present study, an IVDD mouse model was established by excision of the bilateral facet joints and the supraspinous and interspinous ligaments in C57BL/6J male mice, resulting in disc instability and CEP degeneration (Ao et al. 2019). In the IVDD group, CEP degeneration and calcification were observed, characterized by a significant decrease in the deep matrix of the CEP, an increase in bone tissue and a notable rise in the bone marrow content and mineralized bone. To investigate the role of Piezo1 in this process, a Piezo1 agonist and an inhibitor were injected into the IVDD group mice. The results showed that promoting the expression of Piezo1 further aggravated the degeneration and calcification of the CEP. Conversely, inhibiting Piezo1 expression led to an increase in the cartilage matrix of the CEP, a decrease in bone tissue and an increase in the ECM content of both the NP and CEP. These results suggest that manipulating the expression of Piezo1 can influence the degeneration of the CEP and the development of IVDD, providing a novel target for the treatment of IVDD.

To further explore the cellular mechanisms underlying CEP degeneration, primary chondrocytes were isolated as a mixture of growth plate chondrocytes and immature cartilage endplate chondrocytes. Secondary ossification centers begin to form in mice during the second week of life, and while the cartilage endplate gradually becomes isolated from the growth plates, it remains continuous with growth plate cartilage for nearly the entire lifespan of mice. Consequently, isolating pure CEP chondrocytes or growth plate chondrocytes is not feasible. The method employed in this study is well-established and adheres to protocols from prior research (Wang et al. 2024; Wang et al. 2022a, b; Haseeb et al. 2021; Li et al. 2011). Growth plate chondrocytes actively proliferate in the columnar zone of the tissue and undergoing terminal hypertrophic maturation, whereas adult cartilage endplate chondrocytes are non-proliferative and typically do not proceed to terminal maturation. In this study, this method yielded primary chondrocytes with robust growth status, meeting the experimental standards. These cells provided a reliable model to investigate the molecular pathways involved in CEP degeneration.

The degeneration of CEP is influenced by various risk factors, including mechanical overload, chemical exposure, injury, instability, bipedal posture, genetic predisposition, and smoking, which can trigger the release of reactive oxygen species (ROS) and proinflammatory cytokines. This results in oxidative stress and a chondrocyte inflammatory response, which reduces cell viability and promotes CEP degeneration (Vergroesen et al. 2015; Wang et al. 2016; Wang et al. 2021; Dowdell et al. 2017). In the present study, TBHP was used to induce oxidative stress in chondrocytes, which increased the ROS levels and subsequently elevated Piezo1 expression. The results demonstrated that higher oxidative stress levels were closely associated with increased Piezo1 expression. To investigate the mechanism by which Piezo1 induces CEP degeneration and chondrocyte apoptosis, Piezo1 expression was manipulated by drugs or (si)RNA. Enhancing Piezo1 expression increased the ROS levels, oxidative stress, and inflammation in chondrocytes. This activation of the NF-κB pathway resulted in elevated p-p65/p65 and p-IκB/IκB levels, which exacerbate the inflammatory response leading to ECM degradation. This pathway may be Yap independent. Meanwhile, MMP3 and MMP13 expression was upregulated, while SOX9 and COL2 expression was downregulated, further exacerbating the inflammatory response and leading to an increase in chondrocyte apoptosis. Additionally, the chondrocyte hypertrophy and osteogenesis markers, COL10 and RUNX2 were both elevated, indicating that Piezo1 expression promoted calcification of the CEP. By contrast, inhibiting Piezo1 expression mitigated these results. Finally, the findings of the present study indicated that intervening in the expression of Piezo1 could regulate the oxidative stress levels and the inflammatory response in chondrocytes, thereby affecting CEP degradation and the chondrocyte calcification levels, affecting the progression of IVDD.

Voltage-sensitive ion channels (Piezo1 is a force-sensitive ion channel) are cationic channels that regulate apoptosis by regulating Ca^2+^ influx (Coste et al. 2010; Borbiro et al. 2017; Koike et al. 2015; Onitsuka et al. 2020). Piezo1, a member of this family, exhibits increased expression following mechanical stimulation (Chen et al. 2023). RhoA is an essential component of the cytoskeleton and RhoA-mediated F-actin remodeling serves a crucial role in regulating Yap (Xu et al. 2012). BAPTA-AM, a Ca^2+^ channel blocker, restricted intracellular Ca^2+^ influx, which limited cytoskeletal aggregation, RhoA-mediated F-actin remodeling and Yap activation, delaying ECM degradation. Consequently, the MMP3 and MMP13 protein expression levels were weakened and the SOX9 and COL2 protein expression levels enhanced. Furthermore, the inflammation levels were reduced, as indicated by decreased NLRP3 and Caspase-1 expression, and cell calcification was diminished, as evidenced by reduced COL10 and RUNX2 protein expression. Similarly, Latrunculin A, a cytoskeleton inhibitor, was prevented cytoskeleton polymerization, which affected the voltage-sensitive ion channels and weaked the influx of Ca^2+^ in chondrocytes. This leaded to a decrease in the Yap/p-Yap ratio, along with reduced expression of CTGF, thereby slowing the degeneration of the CEP. These results suggested that Ca^2+^ influx and F-actin polymerization played important roles in Piezo1-mediated mechanical signal transduction and CEP degeneration. In addition, previous scholars have summarized that the assembly and activation of NLRP3 are related to Ca^2+^aggregation (Elliott et al. 2015). This study demonstrated that activation of Piezo1 leads to intracellular Ca^2+^ aggregation and an increase in NLRP3 expression; The results are consistent with previous findings, suggesting the possibility of an inflammatory response that is independent of the Yap pathway, which requires further experimental verification.

Yap shuttles between the cytoplasm and the nucleus, can be activated by oxidative stress and participates in chondrocyte degeneration and apoptosis (Xiang et al. 2018). Piezo1 and Yap are both important mechanical stimulators; however, whether Yap mediates Piezo1 activation-induced mechanical signal transduction remains elusive (Puleo et al. 2019; Hasegawa et al. 2021). The results of the present study indicated that inhibiting Piezo1 also inhibited Yap activation. Moreover, Yap inhibition ameliorated CEP chondrocyte apoptosis and ECM degradation and alleviated the inflammatory response, as evidenced by the decreased expression of NLRP3 and Caspase-1. A previous study has shown that Yap serves an important role in osteocyte proliferation and osteogenic differentiation under mechanical stress (Lawrence et al. 2017). In the present study, it was demonstrated that inhibiting Yap expression resulted in reduced chondrocyte hypertrophy and decreased levels of the osteogenic markers, COL10 and RUNX2, thereby slowing IVDD.

## Conclusion

The results of the present study demonstrated that Piezo1 participates in IVDD. Piezo1 contributed to disc degeneration by increasing ROS production in chondrocytes, increasing the oxidative stress level, promoting ECM degradation, triggering the inflammatory response and inducing apoptosis. It was shown that Yap functions downstream of Piezo1 and mediates the Piezo1-induced signal transduction. Piezo1 activation facilitated Ca^2+^ influx and F-actin polymerization, thus promoting Yap activation through the F-actin-mediated non-canonical signaling pathway. Therefore, inhibiting Yap could ameliorate CEP degeneration. The present study provides new insights into the mechanisms driving IVDD and potential therapeutic targets.

## Limitations and perspectives

In this study, GsMTx4 was employed to conduct animal experiments, ALP assays, and alizarin red staining to evaluate the effects of Piezo1 inhibition on the cartilage endplate and chondrocytes. However, it should be noted that GsMTx4 is not a specific inhibitor of Piezo1; it also targets other cationic mechanosensitive channels (MSCs), including TRPC1, TRPC6, and Piezo2. Similarly, verteporfin was used to block the Yap/TEAD interaction to investigate whether Yap exerts its function through binding to TEAD. Nevertheless, verteporfin exhibits off-target effects, such as downregulating oncogenic and pro-survival proteins. Future research in this field could benefit from the use of more specific inhibitors, such as IAG933, to yield more precise and reliable results.

This study did not explore the role of Piezo1 in chondrocytes under varying stress conditions. The intervention experiment using different stresses seems to be more in line with the environment in which chondrocytes live. Additionally, based on our experimental findings, we identified that NLRP3 and Caspase-1 proteins are associated with this pathway, both of which play roles in promoting the release of IL factors. These IL factors can trigger inflammatory responses, which are known contributors to cartilage endplate degeneration. Therefore, we believe it is essential to investigate the specific relationship between this pathway and NLRP3 under different stress conditions. This will be a key focus of our future research, as understanding these interactions could reveal novel therapeutic targets for mitigating cartilage degeneration.

## Data Availability

The datasets used and/or analyzed during the current study are available from the corresponding author on reasonable request.

## Abbreviations

IVDD: Intervertebral disc degeneration
CEP: Cartilage endplate
NP: Nucleus pulposus
ROS: Reactive Oxygen species
ECM: Extracellular matrix
Yap: Yes-associated protein
FA: Focal adhesions
BMD: Bone mineral density
